# Plasma MMP-9, TIMP-1, and TGF-β1 Responses to Exercise-Induced Muscle Injury

**DOI:** 10.3390/ijerph17020566

**Published:** 2020-01-16

**Authors:** Jooyoung Kim, Joohyung Lee

**Affiliations:** 1 Department of Anatomy, School of Medicine, Kyungpook National University, Daegu 41566, Korea; hirase1125@hanmail.net; 2 Clinical Omics Institute, Kyungpook National University, Daegu 41566, Korea; 3 Department of Sport, Health and Rehabilitation, College of Physical Education, Kookmin University, Seoul 02707, Korea

**Keywords:** eccentric exercise, muscle injury, scar tissue, strength recovery, transforming growth factor-β1

## Abstract

The purpose of this study was to analyze changes in the levels of matrix metalloproteinase-9 (MMP-9), tissue inhibitor of metalloproteinase-1 (TIMP-1), and transforming growth factor-β1 (TGF-β1) in response to strength recovery after eccentric exercise in humans. In this study, 16 healthy males participated and were divided into a faster recovery (FR) group (n = 8, >80% of baseline) and a slower recovery (SR) group (n = 8, <40% of baseline) on the basis of their recovery of maximal isometric strength (MIS) 96 h after eccentric exercise. For both groups, measurements were taken of muscle soreness, creatine kinase (CK) activity, and MMP-9, TIMP-1, and TGF-β1 levels during the 24- to 96-h period after eccentric muscle contraction of their non-dominant elbow flexor. Muscle soreness (*p* < 0.001), CK activity (*p* < 0.01), and TGF-β1 level (*p* < 0.01) were significantly lower in the FR group compared with SR group, whereas no significant differences in MMP-9 and TIMP-1 levels were found between the two groups (*p* > 0.05). These results suggest that scar tissue formation caused by the pro-fibrotic activity of growth factors such as TGF-β1 is a potential cause of delay in strength recovery after exercise-induced muscle injury.

## 1. Introduction

High-intensity exercise accompanied by eccentric muscle contractions is known to cause injury to myofibers [1]. In turn, muscle injury can result in loss of maximal strength, increased muscle soreness, and elevation of plasma proteins such as creatine kinase (CK) or myoglobin (Mb) [2]. Of the numerous markers of muscle injury, loss of maximal strength is considered the most reliable and valid indirect marker of muscle [3]. Generally, maximal strength is reduced by 50–60% immediately after eccentric exercise and increases gradually during the recovery period [4].

Several studies have reported an inter-subject variability in strength recovery following eccentric exercise [2,4,5]. Sayers and Clarkson [4] reported that the length of time needed to achieve complete recovery varied among subjects after the initial loss of 57–70% in muscle strength immediately after eccentric exercise. Paulsen et al. [2] also stated that the degree of strength recovery differed among subjects after an immediate loss of strength, ranging from 19% to 73% following eccentric exercise. Besides, Hubal et al. [5] reported a significant difference in strength recovery among subjects who demonstrated a similar decline in muscle strength loss averaging 53% immediately after eccentric exercise.

Meanwhile, the recovery from exercise-induced muscle injury involves sequential multiple events ranging from degeneration, inflammation, and fibrosis to regeneration [6]. With respect to this, several studies have pointed out the formation of scar tissue caused by fibrotic responses as a possible obstacle to recovery from muscle injury [7,8]. Injured muscle is also known to result in increased levels of matrix metalloproteinase-9 (MMP-9) and tissue inhibitor of metalloproteinase-1 (TIMP-1) [9,10]. Subsequently, MMP-9 increases tissue degradation, whereas TIMP-1 suppresses the substrate-degrading function of MMP-9 [11]. While a proper balance between these proteins can promote muscle regeneration and regulate fibrous development, any abnormality in their balance or overactivity can lead to fibrosis [6]. In particular, MMP-9 has the ability to activate transforming growth factor-β1 (TGF-β1) [12]. The expression of TGF-β1 increases in injured muscle, and its overexpression promotes fibrous scar tissue formation through receptor-mediated Smad signaling pathways, leading to a decline in muscle function [13]. An animal study conducted both in vitro and in vivo supported that TGF-β1 inhibitors such as suramin can be administered after a muscle strain injury to reduce scar tissue formation and accelerate the recovery of strength [14]. The use of TGF-β inhibitors also helped to improve functional recovery after eccentric-contraction-induced muscle injury in an in situ study, which is likely due to a restrained accumulation of scar tissue in the injured part [15]. These animal studies demonstrated that inhibited TGF-β levels led to a blunted scar tissue formation, but in humans it is not clear whether levels of TGF-β following muscle injury have a positive or negative effect in strength recovery.

Taken together, eccentric-exercise-induced muscle injury increases the expression levels of MMP-9, TIMP-1, and TGF-β1, with excessive increases of TGF-β1 levels particularly involved in the formation of scar tissue, which has a high possibility of delaying strength recovery. While most studies focused on investigating time-dependent changes in MMP-9, TIMP-1, and TGF-β1 levels after eccentric muscle contraction in both human and animal models [16,17], studies that examined how variations in these protein levels are related to strength recovery are still limited. In this study, we examined the relationships of MMP-9, TIMP-1, and TGF-β1 levels with strength recovery after eccentric exercise in humans. Based on the previous studies, when TGF-β levels are low, less scar tissues were formed, leading to improved strength recovery. Therefore, we hypothesized that MMP-9, TIMP-1, and TGF-β1 levels would be lower in the group with faster strength recovery after eccentric exercise.

## 2. Materials and Methods

### 2.1. Subjects

A total of 35 healthy male college students (age: 20.5 ± 0.8 years, height: 175.2 ± 4.8 cm, weight: 68.1 ± 9.1 kg, body mass index (BMI): 22.1 ± 2.6 kg/m^2^) were initially recruited. All the subjects were briefed about the study procedures, and an informed consent form approved by the University Institutional Review Board was signed by each subject. Subjects were divided into a faster recovery (FR) group (n = 8, >80% of baseline) and a slower recovery (SR) group (n = 8, <40% of baseline), according to their maximal isometric strength (MIS) of the exercised arm measured at 96 h after exercise [18]. Subjects who had MIS between 40% and 80% were not included in the analysis. The characteristics of subjects included in the study and the baseline measures are shown in Table 1. The inclusion criteria consisted of non-smokers; subjects who did not participate in any resistance training during the past 6 months; those who were not taking nutritional supplements such as proteins or vitamins, or anti-inflammatory drugs; and those who had no musculoskeletal diseases. This information was confirmed through interviews and a questionnaire survey conducted before the experiment.

### 2.2. Eccentric Exercise

Each subject performed eccentric exercise on a modified preacher curl machine (EMC model, Kookmin University, Seoul, Korea). At the beginning of the exercise, subjects were asked to sit down on the preacher bench and position their elbow flexor at 90 degrees against the preacher bench pad. Following a starting signal from the investigator, the subject was to flex his elbow to pull the pad up with maximal force, and then the investigator pulled the lever of the preacher curl down in the opposite direction from the subject, inducing eccentric muscle contraction of the elbow flexor muscles. Each eccentric contraction was maintained for three seconds, followed by a 12-s rest period. Two sets of 25 contractions were performed with five-minute rest periods between sets. In this study, the eccentric exercise protocol developed by Clarkson et al. [19] was used for reference.

### 2.3. Maximal Isometric Strength

The MIS of the elbow flexor muscles was assessed using a strain gauge (Jackson Strength Evaluation System Model 32628CTL, Lafayette Instrument Company, Lafayette, IN, USA) that was attached to the modified preacher curl machine. When each subject, sitting on the preacher bench, positioned his non-dominant arm on the pad at 90 degrees and pulled it up towards his chest with maximal force, the MIS was measured three times in total with 30-s rest periods between trials. Each MIS value reported in this study is the average of the three measurements.

### 2.4. Muscle Soreness

Muscle soreness was assessed using a visual analogue scale (VAS) represented by a line of 100 mm length, where 0 mm indicates “no soreness” and 100 mm indicates “severe soreness.” Each subject drew a vertical line on the VAS to indicate the level of muscle soreness that they felt [1].

### 2.5. Creatine Kinase

A 5 mL blood sample was collected from the brachial vein of each subject. The subjects were instructed to fast for 8 h before blood samples were collected. Each blood sample in BD Vacutainer^®^ (SST II Advance Plus Blood Collection Tubes, Becton, Dickinson and Company, Belliver Industrial Estate, Roborough, Plymouth, UK) tube was centrifuged at 2500–3000 rpm for 10 min. The separated plasma was collected into a micro tube (MCT-150-C, Axygen, INC., Union City, CA, USA) and stored in a −80 °C freezer (Ultra Low Temperature Freezer, Operon Co., Ltd., Gimpo, Korea). CK was analyzed using a kit (AceChem CK Kit, YD-Diagnostics Corp., Yongin, Korea) and automated clinical chemistry analyzers (Miura One, I.S.E. S.r.l., Rome, Italy).

### 2.6. Matrix Metalloproteinase-9, Tissue Inhibitor of Metalloproteinase-1, and Transforming Growth Factor-β1

Plasma MMP-9, TIMP-1, and TGF-β1 were analyzed using a kit (ProcartaPlex^®^ Multiplex Immunoassay, Thermo Fisher Scientific, Waltham, MA, USA) in accordance with the procedures suggested by the kit manufacturer. All samples were analyzed using clinical diagnostics instruments (Luminex^®^ 200 System, Luminex Corporation, Austin, TX, USA).

### 2.7. Statistical Analysis

Statistical analysis was performed by using SPSS software (SPSS Statistics 21.0, IBM, Armonk, NY, USA). A repeated-measures analysis of variance was used to analyze group-by-time interaction. If an interaction was found between these factors, a Tukey’s post-hoc test was used. The level of significance was set at *p* < 0.05.

## 3. Results

### 3.1. Changes in MIS in Response to Strength Recovery after Eccentric Exercise

**Figure 1:**
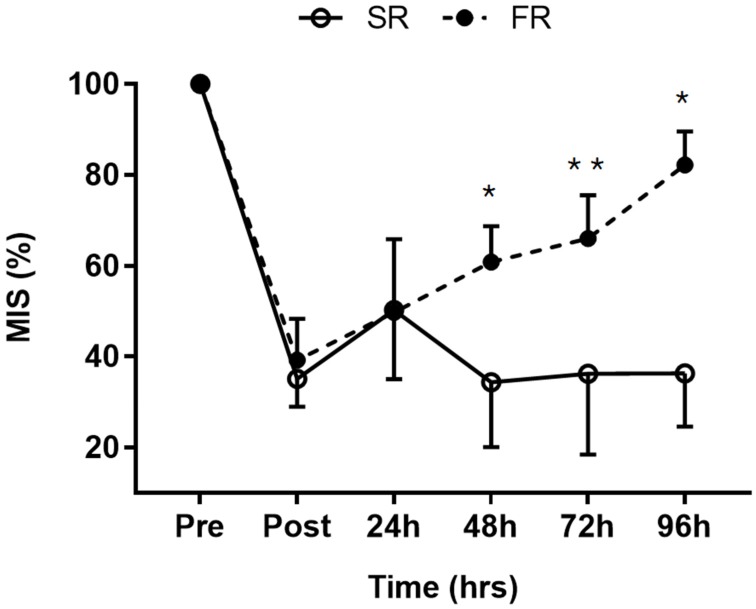
Changes in maximal isometric strength (MIS) in response to strength recovery after eccentric exercise. SR refers to subjects (n = 8) with slower recovery (<40%), and FR refers to subjects (n = 8) with faster recovery (>80%). * Significant between groups (*p* < 0.001), ** Significant between groups (*p* < 0.01). Values are means ± SD.

Significant main effects of time (F = 101.199, *p* < 0.001), group (F = 20.635, *p* < 0.001), and group-by-time interaction (F = 19.319, *p* < 0.001) were observed for MIS. MIS was significantly higher in the FR group than in the SR group at 48 h (*p* < 0.001), 72 h (*p* < 0.01), and 96 h (*p* < 0.001) after exercise (Figure 1). The mean MIS was 82% in the FR group and 36% in the SR group at 96 h after exercise, respectively.

### 3.2. Changes in Muscle Soreness in Response to Strength Recovery after Eccentric Exercise

**Figure 2:**
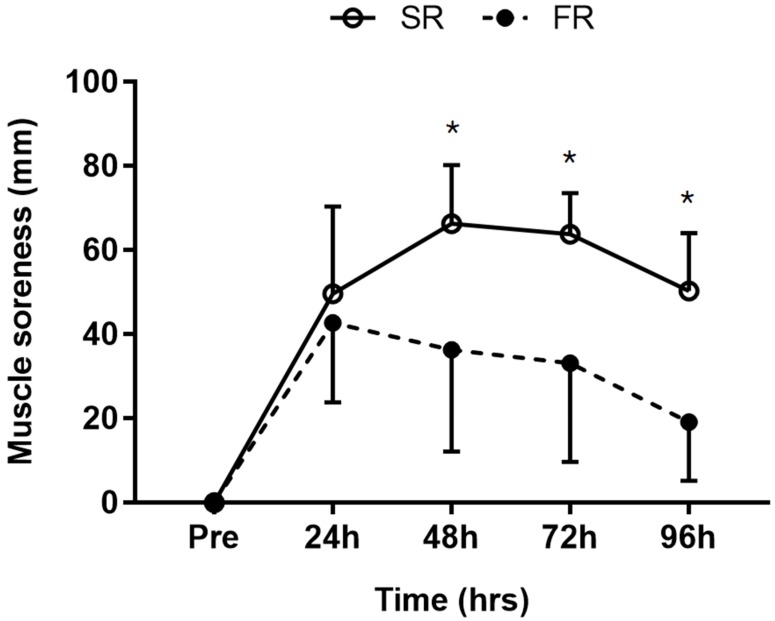
Changes in muscle soreness in response to strength recovery after eccentric exercise. SR refers to subjects (n = 8) with slower recovery (<40%), and FR refers to subjects (n = 8) with faster recovery (>80%). * Significant between groups (*p* < 0.001). Values are means ± SD.

Significant main effects of time (F = 54.914, *p* < 0.001), group (F = 10.047, *p* < 0.01), and group-by-time interaction (F = 6.996, *p* < 0.001) were observed for muscle soreness. Muscle soreness was significantly lower in the FR group than in the SR group at 48, 72, and 96 h after exercise (*p* < 0.001; Figure 2).

### 3.3. Changes in CK Activity in Response to Strength Recovery after Eccentric Exercise

**Figure 3:**
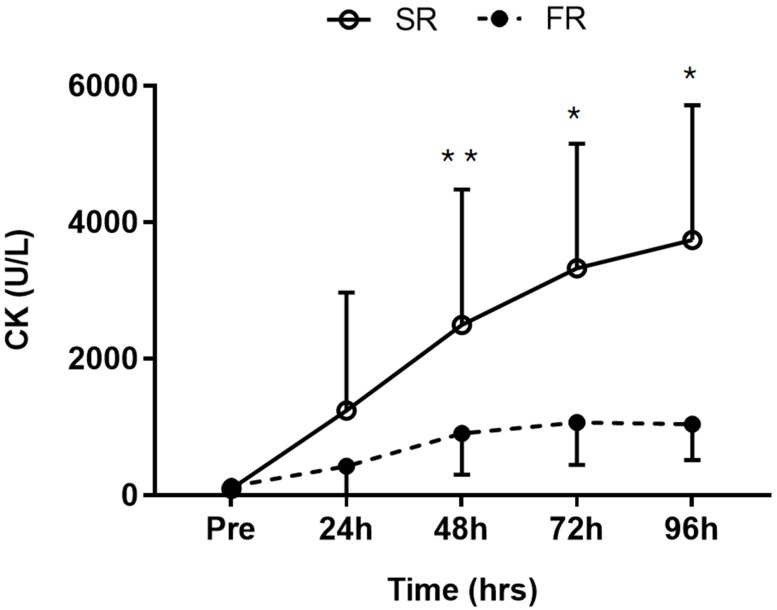
Changes in creatine kinase (CK) activity in response to strength recovery after eccentric exercise. SR refers to subjects (n = 8) with slower recovery (<40%), and FR refers to subjects (n = 8) with faster recovery (>80%). * Significant between groups (*p* < 0.01), ** Significant between groups (*p* < 0.05). Values are means ± SD.

Significant main effects of time (F = 14.360, *p* < 0.001), group (F = 11.886, *p* < 0.01), and group-by-time interaction (F = 4.728, *p* < 0.05) were observed for CK activity. CK activity was significantly lower in the FR group than in the SR group at 48 h (*p* < 0.05), 72 h (*p* < 0.01), and 96 h (*p* < 0.01) after exercise (Figure 3).

### 3.4. Changes in MMP-9 Level in Response to Strength Recovery after Eccentric Exercise

**Figure 4:**
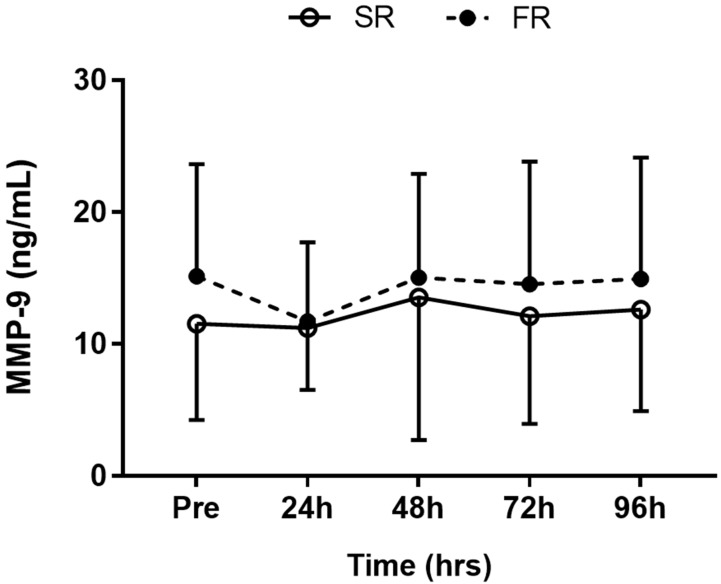
Changes in MMP-9 level in response to strength recovery after eccentric exercise. SR refers to subjects (n = 8) with slower recovery (<40%), and FR refers to subjects (n = 8) with faster recovery (>80%). Values are means ± SD.

No significant main effects of time (F = 1.435, *p* > 0.05), group (F = 0.296, *p* > 0.05), or group-by-time interaction (F = 0.401, *p* > 0.05) were observed for MMP-9 response (Figure 4).

### 3.5. Changes in TIMP-1 Level in Response to Strength Recovery after Eccentric Exercise

**Figure 5:**
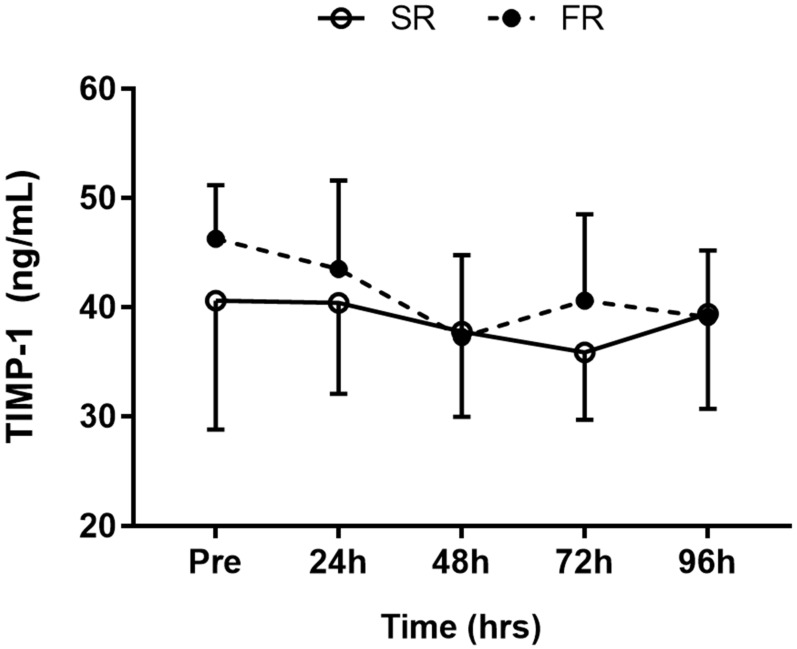
Changes in TIMP-1 level in response to strength recovery after eccentric exercise. SR refers to subjects (n = 8) with slower recovery (<40%), and FR refers to subjects (n = 8) with faster recovery (>80%). Values are means ± SD.

A significant main effect of time (F = 5.315, *p* < 0.001) was observed for TIMP-1 response. However, the main effects of group (F = 0.536, *p* > 0.05) and group-by-time interaction (F = 1.713, *p* > 0.05) were not significant (Figure 5).

### 3.6. Changes in TGF-β1 Level in Response to Strength Recovery after Eccentric Exercise

**Figure 6:**
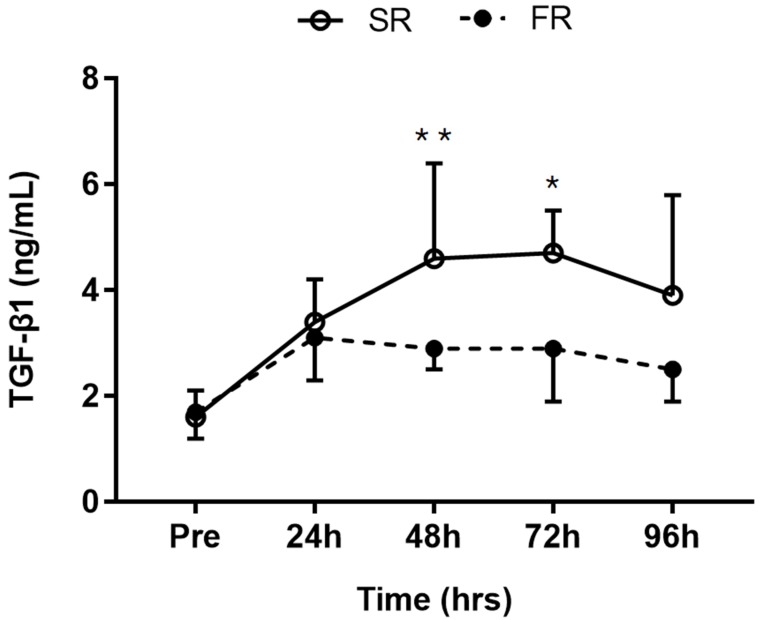
Changes in TGF-β1 level in response to strength recovery after eccentric exercise. SR refers to subjects (n = 8) with slower recovery (<40%) and FR refers to subjects (n = 8) with faster recovery (>80%). * Significant between groups (*p* < 0.01), ** Significant between groups (*p* < 0.05). Values are means ± SD.

Significant main effects of time (F = 15.996, *p* < 0.001), group (F = 7.530, *p* < 0.05), and group-by-time interaction (F = 3.744, *p* <.01) were observed for TGF-β1 response. TGF-β1 level was significantly lower in the FR group than in the SR group at 48 h (*p* < 0.05) and 72 h (*p* < 0.01) after exercise (Figure 6).

## 4. Discussion

This study analyzed the differences in MMP-9, TIMP-1, and TGF-β1 levels between FR and SR groups, divided based on strength recovery following eccentric exercise. Up to 24 h after eccentric exercise, there were no differences in MIS between the two groups, while TGF-β1, muscle soreness, and CK activity increased in both groups, compared to the levels immediately after exercise. However, at 48 h post exercise, the two groups showed completely different patterns. Compared to the SR group, the FR group showed faster recovery in MIS, and TGF-β1 levels, CK activity, and muscle soreness were noted to be lower. It is believed that this was due to scar tissue formation after inflammatory response, which appeared to a lesser degree in the FR group. According to several studies, scar tissue formation due to fibrosis following an acute muscle injury reduces strength recovery [14,15]. It is widely known that fibrotic factors such as TGF-β1 are involved in such scar tissue formation, and some animal studies have reported that a significant increase in TGF-β1 levels after acute muscle injury resulted in increased scar tissue formation in the injured muscles [7,14].

Scar tissue formation can lead to incomplete recovery in the injured muscles due to weakening of the cell membrane and disorganized fiber arrangement, and such changes may act as a cause of continued increase in CK activity [20]. Meanwhile, TGF-β bears a close relevance to the inflammatory response [21]. The inflammatory response after an injury results in the recruitment of neutrophils and macrophages, which may influence the activation and secretion of TGF-β [22]. The muscle soreness measured in this study presents indirect evidence of an inflammatory response after muscle injury [23]. The potential causal agents for this pain are inflammatory factors such as tumor necrosis factor-alpha (TNF-a) and interleukin-1 (IL-1), which can induce nerve growth factor (NGF) [24]. This induced NGF increases the sensitivity of the nociceptors, thus causing an increase in pain [25]. Several studies have reported that a TGF-β-dependent increase in the activity of NGF could function as a latent factor causing pain in chronic pathological conditions [26,27]. NGF has also been correlated to muscle soreness in acute conditions such as eccentric exercises [28,29], and its upregulation after eccentric muscle contraction has been shown to increase muscle soreness [29,30]. Considering this, the high TGF-β1 levels observed in the SR group in the present study could have caused the increase in muscle soreness. Since NGF levels were not measured in this study, further studies in this regard would be beneficial.

In this study, no significant differences were noted in MMP-9 and TIMP-1 levels between the two groups following eccentric exercise. Some studies have reported that significant increases in MMP-9 and TIMP-1 levels were noted after eccentric exercise [9,16], but the values obtained in our study did not statistically differ much from those obtained at resting level. In a study by Madden et al. [31] that used an elbow flexor model for performing eccentric exercise similar to that in our study, no significant changes in MMP-9 and TIMP-1 levels after exercise were reported. It is believed that such conflicting study results may be attributable to differences in the exercise models used. Koskinen et al. [16] and Mackey et al. [9] applied an exercise model that used lower-extremity muscles (downhill running or isokinetic dynamometer). Other studies also reported increased MMP-9 or MMP-9 mRNA levels following marathon or cycling [32,33]. These results demonstrate the possibility of MMP-9 being affected by the activation of large muscle groups. Circulating neutrophil counts and systemic cytokine responses were reported to be higher in eccentric exercise models which use large muscle groups such as downhill running and eccentric cycling (e.g., femoral muscle) than in eccentric exercise models which use small muscle groups (e.g., elbow flexor) [34]. Neutrophils synthesize MMP-9 [35], and systemic cytokines are involved in the activity of MMP-9 [36]. TIMP-1 is a factor that appears together with MMP-9 response [37], and it may have been affected by the same cause.

This study had some limitations. Firstly, scar tissue formation in injured muscles following eccentric exercise was not observed by histological examination. For example, phenomena associated with scar tissue formation such as collagen accumulation or fibroblast proliferation were not identified directly by electron microscopy. In this study, scar tissue formation was estimated with changes in TGF-β1 levels alone. Secondly, indicators of inflammation after injury were not measured. The inflammatory response plays a significant role in causing fibrosis that occurs after muscle injuries [38], and inflammatory cytokines such as TNF-a, interleukin-4 (IL-4), and interleukin-13 (IL-13) are known to contribute to fibrosis [39]. Measuring the activation levels of the cytokines, which may act as indicators of the inflammatory responses, could facilitate a better interpretation of the changes in the TGF-β1 levels, as observed in this study.

Finally, changes following eccentric exercise were observed only up to 96 h post exercise. Gumucio et al. [15] reported that the inhibition of TGF-β after an injury was rapid during the early stage of strength recovery (3–7 days), but after 21 days, strength was actually higher in the control group with no inhibition of TGF-β. Such results indicate that TGF-β can slow the initial recovery process in the injured muscles by scar tissue formation, but it actually has the potential to make the muscles stiffer eventually. Because this study only examined strength recovery up to 96 h post exercise, the similarity in subsequent changes between this study and that by Gumucio et al. [15] could not be determined. Therefore, future studies should address these limitations and conduct more detailed investigations of the effects and roles of TGF-β1 on strength recovery following exercise-induced muscle injury.

## 5. Conclusions

This is the first study showing that strength recovery after eccentric muscle contractions may depend on the levels of TGF-β1 related to scar tissue formation, although other factors could be involved in the process of fibrosis during muscle recovery. Indeed, TGF-β1 levels appeared higher in the group with slower strength recovery after eccentric exercise in this study. These results suggest that scar tissue formation from pro-fibrotic activity of growth factors such as TGF-β1 has the potential to delay strength recovery after exercise-induced muscle injury.

## Figures and Tables

**Table 1:** Subject characteristics and baseline measurements (n = 16, mean ± SD).

	SR (n = 8)	FR (n = 8)
Age (years)	20.0 ± 0.5	20.2 ± 0.8
Height (cm)	175.3 ± 5.1	176.6 ± 4.0
Weight (kg)	67.3 ± 13.1	66.3 ± 5.2
BMI (kg/m^2^)	21.8 ± 3.7	21.2 ± 0.9
CK (U/L)	97.1 ± 59.2	132.5 ± 48.7
MMP-9 (ng/mL)	11.5 ± 7.3	15.1 ± 8.5
TIMP-1 (ng/mL)	40.6 ± 11.8	46.3 ± 4.9
TGF-β1 (ng/mL)	1.7 ± 0.5	1.6 ± 0.5

SR, slower recovery; FR, faster recovery; BMI, body mass index; CK, creatine kinase; MMP-9, matrix metalloproteinase-9; TIMP-1, tissue inhibitor of metalloproteinase-1; TGF-β1, transforming growth factor-β1. There were no significant differences between groups.
